# Identifying Patterns of FEES-Derived Swallowing Trajectories Using Group-Based Trajectory Model

**DOI:** 10.1007/s00455-015-9632-3

**Published:** 2015-07-25

**Authors:** Laura W. J. Baijens, Walmari Pilz, Bernd Kremer, Valeria Lima Passos

**Affiliations:** Department of Otorhinolaryngology, Head and Neck Surgery, Maastricht University Medical Center, PO Box 5800, 6202 AZ Maastricht, The Netherlands; Department of Methodology and Statistics, Maastricht University, Maastricht, The Netherlands

**Keywords:** Dysphagia, Aspiration, Fiberoptic endoscopic evaluation of swallowing, Deglutition, Deglutition disorders, Group-based trajectory modeling

## Abstract

The present study delineates and visualizes swallowing trajectories along seven swallow trials in dysphagic patients using group-based trajectory modeling (GBTM). This model facilitates the recognition of swallowing functional categories, estimates their frequency of occurrence, and enhances the understanding of swallowing dynamics. Two hundred and five dysphagic patients underwent a standardized FEES examination protocol. Five ordinal variables were blindly assessed for each swallow by two observers independently. GBTM analysis was conducted to find and characterize trajectories of FEES responses. For most FEES outcome variables, trajectories were qualitatively distinct in degree and kind (level of impairment and how this changed over the seven swallow trials). Two FEES outcome variables—delayed initiation of the pharyngeal reflex and postswallow pyriform sinus pooling—showed the highest prevalence of severe swallowing impairment. Highly impaired categories were more stable throughout the different swallow trials. Intermediate trajectories, by contrast, were erratic, responding more sensitively to shifts in bolus consistency. GBTM can identify distinct developmental trajectories of measured FEES variables in patients with oropharyngeal dysphagia. In clinical practice, classification into distinct groups would help to identify the subgroup of dysphagic patients who may need specific medical attention.

## Introduction

Fiberoptic endoscopic evaluation of swallowing (FEES) is a reliable tool that allows the dysphagia professional to evaluate the pharyngeal phase of swallowing [1]. FEES is well tolerated, easily repeatable, and can be performed at the bedside [1]. During a standardized FEES examination, patients swallow a sequence of boluses of different consistencies in consecutive order [2, 3]. Usually visuoperceptual ordinal-scale variables are applied to judge FEES images.

FEES data on dysphagic patients are highly heterogeneous, both within and between subjects. This variability is partly due to the diversity of the dysphagic population, reflecting different etiologies of the swallowing dysfunction [Parkinson’s disease (PD), stroke, myopathies, head and neck cancer (HNC), etc.]. However, even within a single etiological group, variability can be substantial. For instance, PD, which is characterized by progressive neuro-degeneration, covers a large group of patients with different levels of disease severity [2]. Therefore, acknowledging potential reasons for the high variability in FEES response (as recorded by repeated measures over the seven swallows) calls for a different analytical approach, one that capitalizes on the heterogeneity of these responses. In this respect, an alternative approach may shed new light on the dynamics of patients’ swallowing capabilities. The alternative presented here allows discernible patterns of swallowing courses to be extracted from the FEES responses.

Group-based trajectory modeling (GBTM) was developed to identify groups of individuals following a similar progression of a certain behavior in a longitudinal setting. This model-based clustering method is often referred to as a *person*-*centered* approach [4–6]. It enables researchers to understand how life-course experiences unfold at the individual level and to cluster individuals who share similar developmental patterns. In GBTM, the population of interest is assumed to be heterogeneous—a mixture of groups. Shi et al. applied GBTM to describe the heterogeneity of symptom burden among patients with HNC and to identify subgroups with distinct symptom development trajectories [7]. Treatment-related symptom burden varies significantly among patients undergoing radiotherapy or chemoradiotherapy, yet such variation is typically not reflected in the results from single-group studies. A two-group GBTM model identified 68 % of patients as having high symptom burden, associated with older age, worse baseline performance status, and chemoradiotherapy treatment [7]. Another example of how GBTM has been used in the past is the study by Pines et al. [8]. They described sexual risk trajectories among HIV-negative men who have sex with men. Three sexual risk trajectory groups were identified: low-risk, moderate-risk, and high-risk sexual behavior. The trajectories were significantly associated with earning an income, distress/depression symptoms, and substance use [8].

However, a FEES examination protocol is of short duration, representing only a snapshot of a patient’s swallowing functional state. Therefore, GBTM is used here primarily for exploratory purposes. The main objective was to identify subgroups of patients with qualitatively distinct responses over the seven swallowing trials. This would allow us to describe the swallowing trajectories’ level, shape, and prevalence and then link these features to the etiology of dysphagia. To our knowledge, GBTM has not yet been considered for analysis of FEES data.

## Materials and Methods

### Participants

Patients were consecutively enrolled in this prospective study while visiting the outpatient clinic of the Maastricht University Medical Center (MUMC) for their dysphagic complaints. Their data were collected as part of the regular healthcare program for oropharyngeal dysphagia (daily clinical practice) [9]. Incoming patients with oropharyngeal dysphagia could be divided into three main diagnostic groups. Dysphagia in the first group was due to HNC and possible oncological treatment effects on swallowing. Dysphagia in the second group was accompanied by PD. In the final group, it was due to myotonic dystrophy type 1 (DM1). Other etiologies of oropharyngeal dysphagia (stroke, Zenker diverticulum, cervical spine degeneration, etc.) were significantly underrepresented in the patient population over the enrollment period (2012–2014) and were not included. During the patient interview, all subjects reported subjective clinical complaints of oropharyngeal dysphagia ranging from mild to severe. These included, among others, slow eating due to prolonged bolus transit times, oropharyngeal passage disorder, coughing while drinking, and choking on foods. All patients were able to perform a swallow on command. The following exclusion criteria were applied: a Mini Mental State Examination (MMSE) score below 23 [10]; concurrent HNC and a neurological disease (or neurosurgical brain intervention); head and neck oncological treatment less than 3 months previously; surgery of the head and neck swallowing region in patients with PD or DM1; extreme fatigue or weakness (unable to sit upright); an unstable period of PD (periods with large fluctuations, especially in motor function); not the same medication regimen for the past 6 weeks in neurological patients (e.g., with PD); and a total laryngectomy.

### FEES Examination Protocol

All patients underwent a standardized examination protocol including a clinical otorhinolaryngological examination by a laryngologist, a clinical observation of oral intake by a speech and language pathologist, a FEES examination, and the MMSE [9, 10]. During the FEES examination, the participants were offered three trials of thin and three trials of thick liquid followed by one small bite-sized cracker (making a total of seven swallow trials). Each liquid trial contained 10 cc of water or applesauce and was dyed with five percent methylene blue. The tip of the flexible fiberoptic endoscope Pentax FNL-10RP3 (Pentax Canada Inc., Mississauga, Ontario, Canada) was positioned just above the epiglottis in what is called the high position [1]. FEES images were obtained using an Alphatron Stroboview ACLS camera, Alphatron light source, and IVACX computerized video archiving system (Alphatron Medical Systems, Rotterdam, The Netherlands) and recorded on a DVD. Neither a nasal vasoconstrictor nor a topical anesthetic had been administered to the nasal mucosa. All examinations were performed during the “on” motor phase in the PD patients (within 90–120 min after the intake of antiparkinsonian medication) [11].

### FEES Outcome Variables

Visuoperceptual ordinal variables were scored for each swallow trial at varying speed (slow motion, normal, up to frame-by-frame speed) (Table 1) [2, 3, 12]. Before assessment of the swallowing acts, two experts received consensus training for these ordinal variables. The protocol of this training has been described in previous studies [2, 3, 12]. The judges were blinded to the etiological group and to each other’s ratings (independent rating). The swallow trials of all participants were scored in randomized order. To obtain intra-observer agreement, each observer performed repeated measurements (again blinded) of all visuoperceptual FEES variables during the second and third swallow of each bolus consistency for twenty randomly selected participants. This was done within a period of 2 weeks. Furthermore, observers were advised to limit the duration of the measurement sessions (max. 2 h per session) to avoid fatigue and prevent instability of observers’ characteristics.

**Table 1:** Description and observer agreement levels for the measured FEES variables

FEES outcome variable	Description	Scale^a^	Inter-observer agreement^b^
Piecemeal deglutition	Sequential swallowing on the same bolus	Five-point scale (0–4) 0 = no additional swallows 1 = one additional swallow 2 = two additional swallows 3 = three additional swallows 4 = four or more additional swallows	Almost perfect agreement for all etiologies (HNC, PD, DM1) Kappa 0.81–0.99
Delayed initiation of the pharyngeal reflex	Delayed onset of the pharyngeal triggering	Three-point scale (0–2) 0 = no delay 1 = head of bolus in valleculae before initiation of pharyngeal reflex 2 = head of bolus in pyriform sinuses or lower before initiation of pharyngeal reflex	Substantial agreement for PD and DM1 Kappa 0.61–0.80
Postswallow vallecular pooling	Pooling in the valleculae after the swallow	Three-point scale (0–2) 0 = no pooling 1 = filling of less than 50 % of the valleculae 2 = filling of more than 50 % of the valleculae	Almost perfect agreement for all etiologies (HNC, PD, DM1) Kappa 0.81–0.99
Postswallow pyriform sinus pooling	Pooling in the pyriform sinuses after the swallow	Three-point scale (0–2) 0 = no pooling 1 = trace to moderate pooling 2 = severe pooling up to complete filling of the sinuses	Substantial agreement for HNC and DM1 Kappa 0.61–0.80
Penetration–aspiration	Penetration or aspiration	Three-point scale (0–2) 0 = no penetration 1 = penetration 2 = aspiration	Almost perfect agreement for all etiologies (HNC, PD, DM1) Kappa 0.81–0.99

<0 less than chance agreement

0.01–0.20 slight agreement

0.21–0.40 fair agreement

0.41–0.60 moderate agreement

0.61–0.80 substantial agreement

0.81–0.99 almost perfect agreement

*HNC* head and neck cancer, *PD* Parkinson’s disease, *DM1* myotonic dystrophy type 1

^a^Lower scores refer to normal functioning whereas higher scores refer to more severe disability

^b^Kappa agreement (linear weighted kappa coefficient)

### Statistical Analyses

#### Descriptive Statistics

Clinical and demographic variables are presented as means and standard deviation (SD) for continuous scales or as absolute numbers and proportions for categorical scales for each etiological category. Comparisons among etiological groups (HNC, PD, and DM1) were conducted with independent samples *t* test or Chi square/Fisher’s exact tests. Intra- and inter-observer agreement was calculated using a linear weighted kappa coefficient.

#### Group-Based Trajectory Modeling (GBTM)

GBTM analysis was conducted to find and characterize swallowing courses (trajectories) for each measured FEES variable. Once such trajectories are identified, they can be described in terms of composition (size of the cluster), level of impairment (e.g., high, low), and how the impairment changes over the swallows (shape and nature of change: stable, erratic, improving, deteriorating, etc.). Given the way the FEES protocol is designed, with a fixed order of bolus consistencies, it is important to keep in mind that the interpretation of the trajectory’s shape indicates how the swallowing function may be affected by the bolus consistencies.

#### Model Fitting, Selection, and Adequacy

The measured FEES variables were analyzed with *‘proc traj,’* an SAS macro developed by Nagin et al. [4–6]. All outcomes with three categories (three-point-scale ordinal variables) were dichotomized prior to statistical analysis. Categories ‘1’ and ‘2’ were changed into a new category, ‘1+,’ indicating impaired swallowing. The category ‘0’ was unchanged and represented normal swallowing. For the variable penetration-aspiration, dichotomization of the data was carried out by collapsing normal and penetration into category ‘0.’ Aspiration was represented by category ‘1+.’ The outcome with five categories (five-point-scale ordinal variable piecemeal deglutition), though being ordinal at the manifest level, was analyzed as a continuous variable. With *proc traj*, the trajectories’ levels and shapes are determined by the model’s regression parameters. Specifically, each latent trajectory can be characterized by a starting value of impairment level (*intercept*) and possibly by a polynomial function (*linear, quadratic, cubic*), thereby capturing the start level and the shape of the developmental course, respectively. The parameters were estimated by maximum likelihood, with the link functions ‘logit’ for dichotomized FEES variables and ‘censored normal’ for piecemeal deglutition, respectively.

Given the relatively small sample size, the maximum number of clusters considered was five (*k* = 5) and the highest polynomial order was cubic. Model fitting was conditioned on the risk factor etiology. Model selection was conducted in a two-step procedure, according to recommended guidelines [4–6]. Starting with the highest number of clusters (*k* = 5), each with a cubic polynomial trend, k was decreased until a model with the best Bayesian Information Criterion (smallest BIC) was obtained. Polynomial regression coefficients were selected on the grounds of statistical significance of the polynomial terms and BIC. Individuals’ cluster assignment was based on the maximum posterior probability (PP) of cluster membership, as estimated by the selected model. Model adequacy was checked by means of the average posterior probability (APP) of each cluster (desirable average APP ~0.7).

## Results

### Observers’ Agreement

The intra-observer agreement was sufficient for all FEES variables (kappa ≥ 0.61). For some FEES variables (delayed initiation of the pharyngeal reflex and postswallow pyriform sinus pooling), however, the inter-observer agreement was insufficient when calculated per etiological subgroup (HNC, PD, DM1) (Table 1).

### Demographic and Clinical Characteristics

**Fig. 1:**
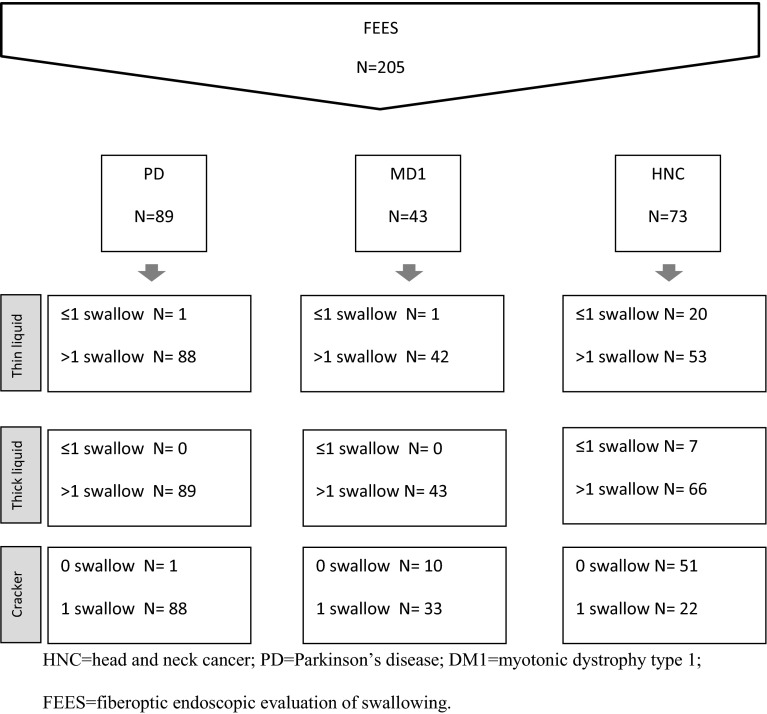
Flowchart of the number and etiology of the included dysphagic patients and the number of swallow trials per patient group and per consistency

Demographic and clinical characteristics are presented in Table 2 for each etiological subgroup. Two hundred and five patients were enrolled in this study. The flow diagram (Fig. 1) displays the distribution of patients over the swallow trials according to their dysphagia etiology. Not everyone could complete all trials at each consistency, so some values are missing due to various causes. For example, HNC patients with severe xerostomia or without a dental prosthesis were not able to perform trials with a dry bite-sized cracker, so these values are reported as missing. Other patients exhibited more severe aspiration with thin liquids, resulting in less than three swallow trials for this consistency. Several DM1 patients performed six swallow trials but did not complete the sequence due to fatigue. It should be noted that data were obtained for all etiological groups (HNC, PD, and DM1: *N* = 205) for piecemeal deglutition, postswallow vallecular pooling, and penetration-aspiration. For delayed initiation of the pharyngeal reflex and postswallow pyriform sinus pooling, the sample sizes were reduced to 116 (HNC and DM1) and 132 subjects (PD and DM1), respectively. The inter-observer agreement for these measured variables was not sufficient for all etiological groups (Table 1).

**Table 2:** Patients’ demographic and clinical characteristics

	HNC (*N* = 73)		PD (*N* = 89)		DMI (*N* = 43)	
Male Female	59 14		66 23		27 16	
	Median (25th, 75th perc)	Range	Median (25th, 75th perc)	Range	Median (25th, 75th perc)	Range
Age	67 (60, 73)	30-83	67 (61, 73)	42-88	46 (38, 55)	21–69

*HNC* head and neck cancer, *PD* Parkinson’s disease, *DM1* myotonic dystrophy type 1, *perc* percentile

### GBTM Results

**Fig. 2:**
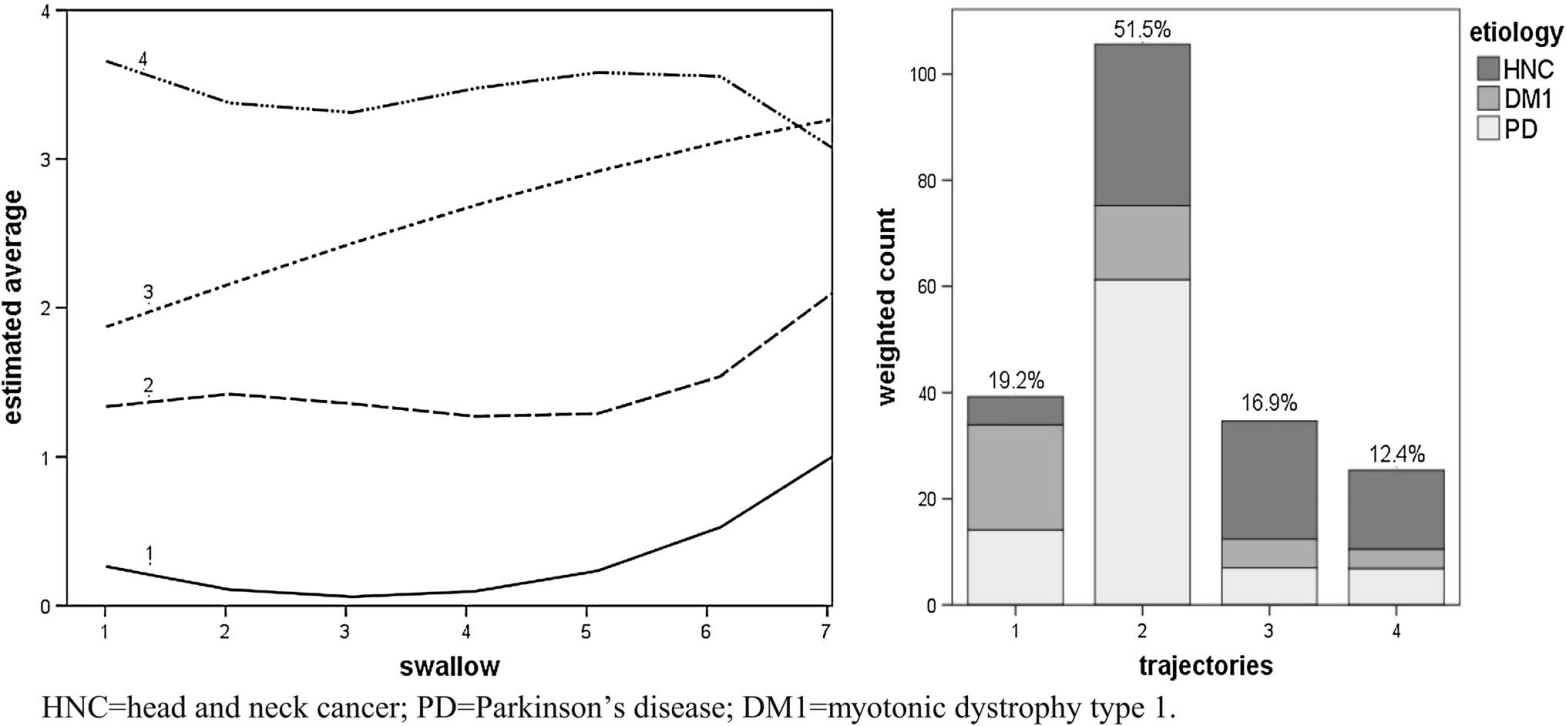
Four swallow trajectories of the FEES variable piecemeal deglutition (*1* low impairment, *2–3* intermediate impairment, *4* high impairment). The *y axis* represents the estimated average score. The *x axis* represents the number of swallow trials and their consistency (*1–3* thin liquid, *4–6* thick liquid, *7* bite-sized cracker) (*left figure*). The *bar charts* (in the *right figure*) present the estimated prevalence (%) of the swallowing trajectories together with their etiology distribution

**Fig. 3:**
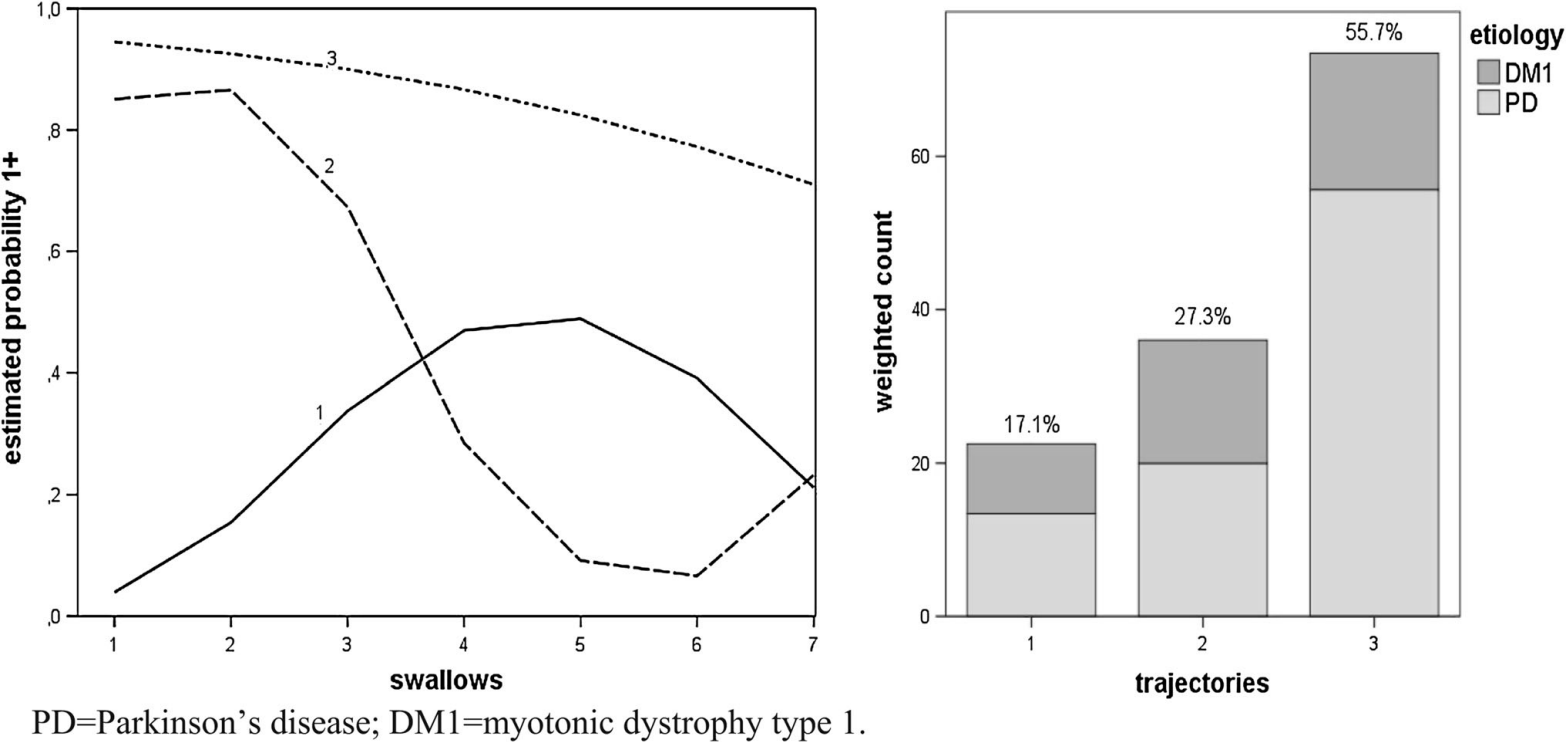
Three swallow trajectories of the FEES variable delayed initiation of the pharyngeal reflex (*1* low impairment, *2* intermediate impairment, *3* high impairment). The *y axis* represents the estimated probability of being classified in impaired categories (equal to 1+). The *x axis* represents the number of swallow trials and their consistency (*1–3* thin liquid, *4–6* thick liquid, *7* bite-sized cracker) (*left figure*). The *bar charts* (in the *right figure*) present the estimated prevalence (%) of the swallowing trajectories together with their etiology distribution. Note: The *height of the bar* indicates the number of patients, weighted by their posterior probability of assignment to the respective trajectory

**Fig. 4:**
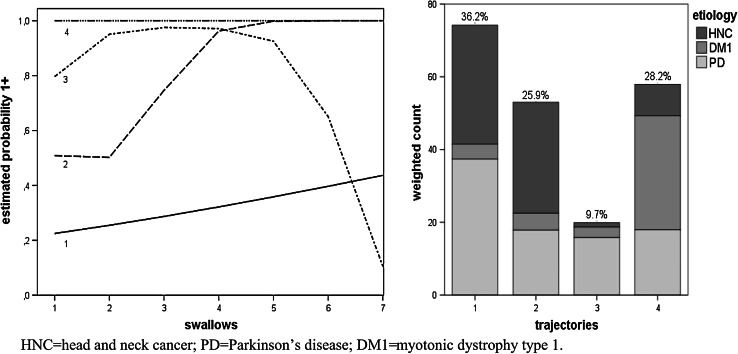
Four swallow trajectories of the FEES variable postswallow vallecular pooling (*1* low impairment, *2–3* intermediate impairment, *4* high impairment). The *y axis* represents the estimated probability of being classified in impaired categories (equal to 1+). The *x axis* represents the number of swallow trials and their consistency (*1–3* thin liquid, *4–6* thick liquid, *7* bite-sized cracker) (*left figure*). The *bar charts* (in the *right figure*) present the estimated prevalence (%) of the swallowing trajectories together with their etiology distribution

**Fig. 5:**
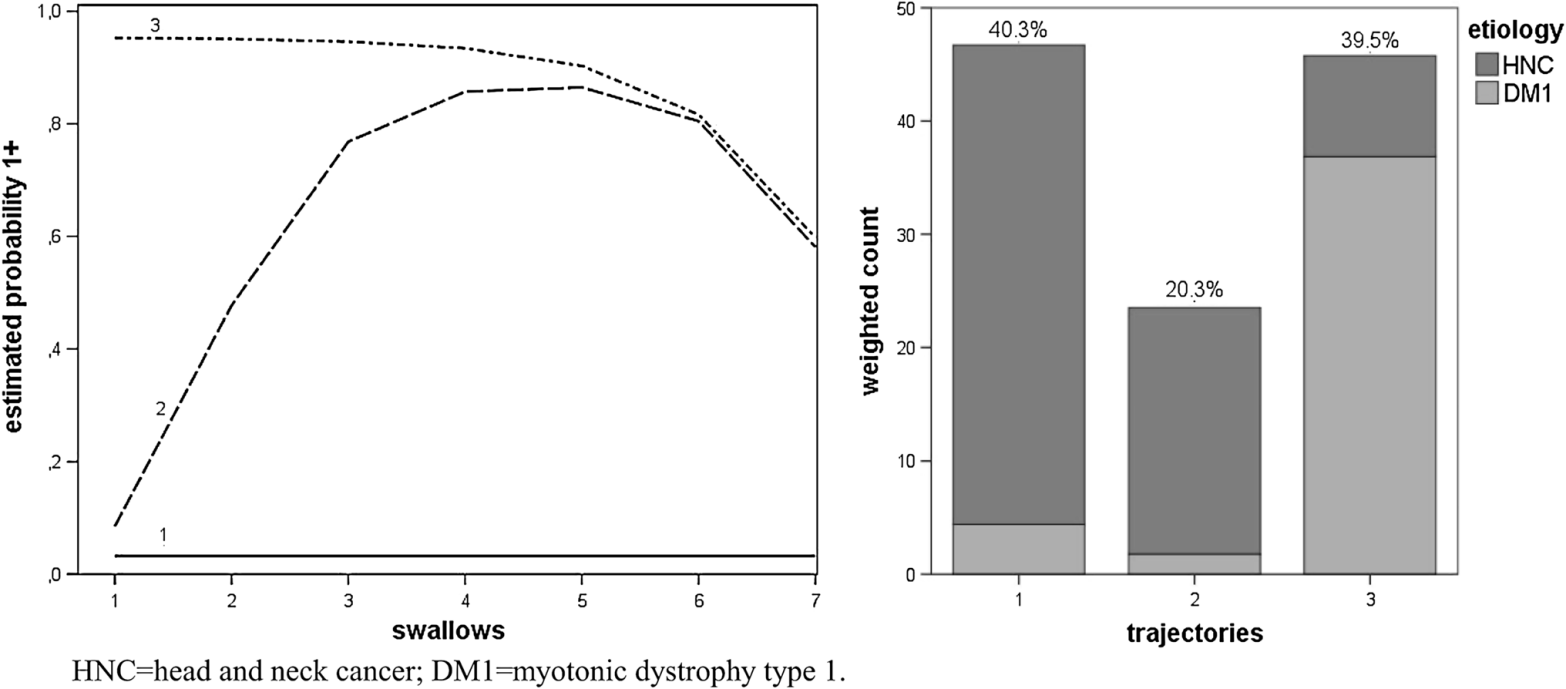
Three swallow trajectories of the FEES variable postswallow pyriform sinus pooling (*1* low impairment, *2* intermediate impairment, *3* high impairmentent). The *y axis* represents the estimated probability of being classified in impaired categories (equal to 1+). The *x axis* represents the number of swallow trials and their consistency (*1–3* thin liquid, *4–6* thick liquid, *7* bite-sized cracker) (*left figure*). The *bar charts* (in the *right figure*) present the estimated prevalence (%) of the swallowing trajectories together with their etiology distribution

**Fig. 6:**
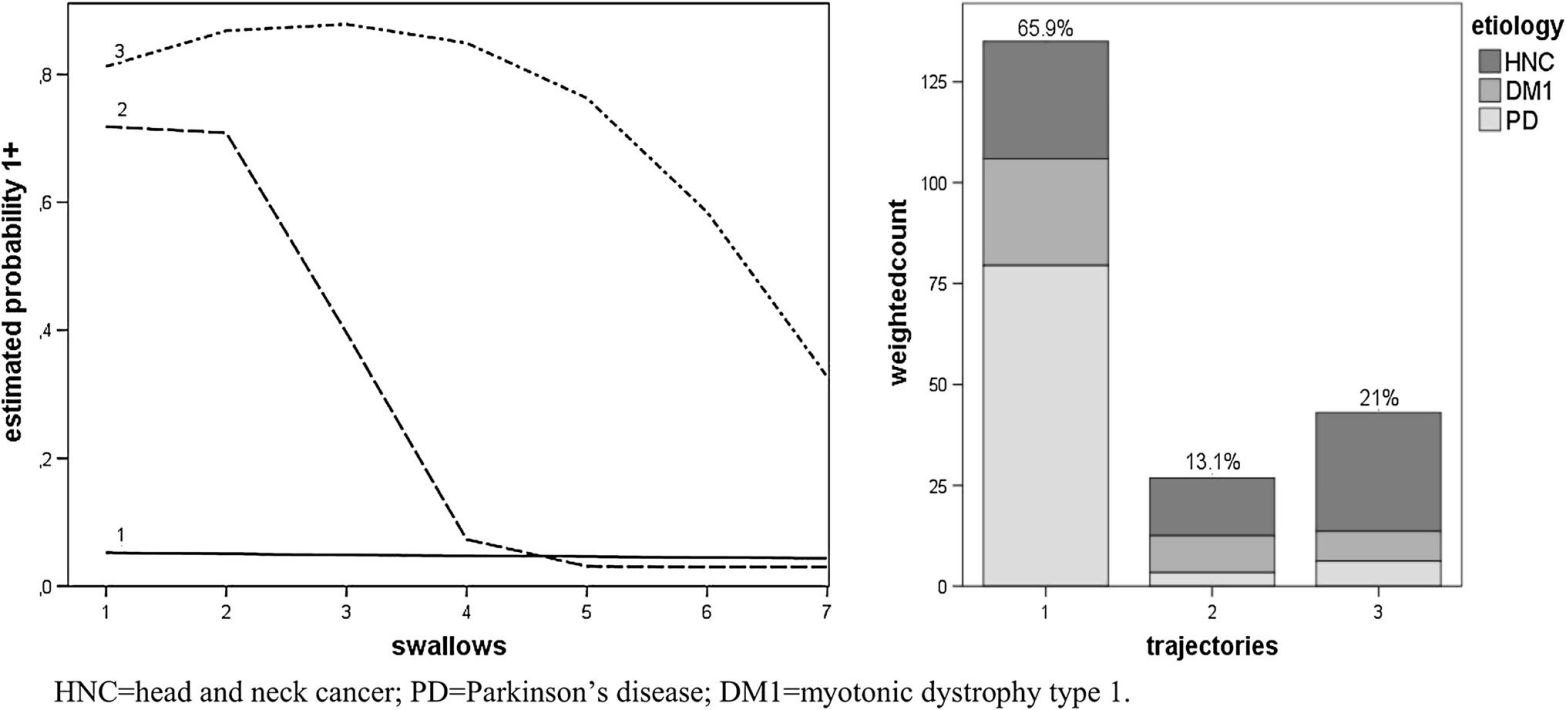
Three swallow trajectories of the FEES variable penetration-aspiration (*1* low impairment, *2* intermediate impairment, *3* high impairment). The *y axis* represents the estimated probability of being classified in impaired categories (equal to 1+). The *x axis* represents the number of swallow trials and their consistency (*1–3* thin liquid, *4–6* thick liquid, *7* bite-sized cracker) (*left figure*). The *bar charts* (in the *right figure*) present the estimated prevalence (%) of the swallowing trajectories together with their etiology distribution

Figures 2, 3, 4, 5 and 6, which illustrate the GBTM output, display the estimated level and shape of the identified FEES trajectories. They show how the probability of being classified in impaired categories (equal to 1+) changed over the seven swallow trials. For the variable piecemeal deglutition, changes in averages over the swallow trials are displayed instead of probabilities (Fig. 2). The mixture proportions (in %, representing the estimated size of the latent cluster) are given together with the etiology distribution per trajectory (bar charts). There, the height of the bar indicates the count of subjects assigned to the trajectory, weighted by their respective posterior probabilities.

A great diversity in swallowing behavior was observed for all measured FEES variables. For most of them, the trajectories were qualitatively distinct, both in degree and kind. In general, the bottom and top FEES trajectories (usually 1 and 3 or 4), reflecting low and high impairment in swallowing function, were less erratic than the intermediate ones and thus less sensitive to changes in bolus consistency. These trajectories for all measured FEES variables can be seen as ‘lines’ of pulled data of all the patients, regardless of their diagnosis (HNC, PD, and DM1).

For piecemeal deglutition (Fig. 2), four trajectories were identified. They were rather stable (flat), differing more in level of impairment than in their shapes/courses. The exception was trajectory no. 3, which steadily deteriorated over the swallows. In general, the swallowing function is more impaired with a bite-sized cracker. The etiology distributions over the trajectories are given in the bar charts of Fig. 2. Relative to the bottom trajectory (1) and taking the PD group as a reference, HNC patients were more frequently assigned to the impaired trajectories 3 (OR_HNC_ = 8.41 with 95 % CI [1.62, 43.65]) and 4 (OR_HNC_ = 5.75 with 95 % CI [1.10, 29.85]). DM1 patients, by contrast, tended to cluster around the lower trajectories, being significantly less likely than PD patients to fall in trajectory 2 (OR_DM1_ = 0.16 with 95 % CI [0.05, 0.47]).

For one variable, delayed initiation of the pharyngeal reflex (PD and DM1, only), swallowing function improved as bolus consistency changed into thick liquid and bite-sized cracker, either gradually (trajectory no. 3) or abruptly (trajectory no. 2) (Fig. 3). For the cluster of patients in trajectory no. 1, swallowing function decreased as bolus consistency changed into thick liquid (Fig. 3). Relatively many patients were assigned to the most impaired category (bar chart Fig. 3). Etiologies were similarly distributed over the three trajectories.

In Fig. 4 (postswallow vallecular pooling), the bottom trajectory (low impairment) had the highest prevalence (36.2 %). Note that for these patients, a slow and gradual deterioration was observed over the swallow trials. The intermediate trajectory no. 3 fared badly with liquids (high probability of impairment) and abruptly changed for the better with the swallow of a bite-sized cracker. Patients assigned to trajectory no. 2 showed the opposite behavior, deteriorating considerably as the bolus consistency changed into thick liquid and a bite-sized cracker. DM1 patients had significantly more chance to be assigned to the most stable and highly impaired trajectory no. 4 compared to PD subjects (OR_DM1_ = 15.79 with 95 % CI [4.41, 56.48]) (Fig. 4).

For the variable postswallow pyriform sinus pooling (DM1 and HNC only), the bottom trajectory, with the lowest impairment, had the highest prevalence (40.3 %) (Fig. 5). The intermediate trajectory no. 2 abruptly changed for the worse when thick liquid swallow trials were given, with a minor recovery during ingestion of a bite-sized cracker. HNC patients had significantly less chance to be assigned to the highly impaired trajectory no. 3 compared with DM1 patients (OR_DM1_ = 0.02 with 95 % CI [0.005, 0.11]) (Fig. 5).

The vast majority of patients (~66 %) had a low probability of aspiration over all swallow trials (Fig. 6). The rest were distributed over two distinct patterns of aspiration (trajectories 2 and 3). Patients assigned to trajectory no. 2 only showed a high probability of aspiration with thin liquid, their performance clearly improving with more solid consistencies. Patients in trajectory no. 3, by contrast, were clearly susceptible to aspiration, improving slightly with bite-size crackers. Etiology distributions differed significantly among the trajectories. DM1 and HNC patients were more likely to be assigned to trajectory no. 2 compared to PD patients, with OR_DM1_ = 8.08, 95 % CI [1.45, 44.92] and OR_HNC_ = 11.59, 95 % CI [2.17, 61.79]. The ORs for trajectory no. 3 were OR_DM1_ = 3.56, 95 % CI [1.03, 12.19] and OR_HNC_ = 12.93, 95 % CI [4.71, 35.49].

## Discussion

Heterogeneity of FEES outcomes makes it difficult for practitioners to pinpoint and characterize regular or irregular patterns of swallowing behavior in population-based studies. There is great demand for evidence-based data that could enhance the clinicians’ interpretation, justify generalizations, and support potential clinical decisions.

This study confirms the hypothesis that GBTM can identify (latent) subgroups of developmental courses for measured FEES variables. Its application uncovered the variability of onset and demonstrated the reactive developmental changes as a function of the swallow trials. This information, in turn, facilitated the recognition of typical or atypical swallowing behaviors, allowing an estimation of their prevalence. The link of the trajectories to the etiology of oropharyngeal dysphagia was found to be significant for most FEES variables. For instance, DM1 patients tend to fall in the higher, impaired trajectories for the variables postswallow vallecular pooling and postswallow pyriform sinus pooling, whereas the opposite applies to the variables piecemeal deglutition and penetration-aspiration [3]. This finding is consistent with a previous study showing more severe impairment for postswallow vallecular pooling when swallowing thick liquid compared with thin. DM1 patients performed better (i.e., had less impaired FEES outcomes) with the solid bolus consistency (bite-sized cracker) than with the other consistencies for the variable postswallow vallecular pooling [3].

HNC patients, by contrast, fell in less impaired trajectories for the variables postswallow vallecular pooling and postswallow pyriform sinus. They had a higher risk of being assigned to top, impaired trajectories for the variables piecemeal deglutition and penetration-aspiration compared to DM1 patients [3, 13]. This makes sense, as (chemo) radiation therapy for HNC can cause sensory impairment, xerostomia, and fibrosis of the upper aerodigestive tract, any of which could increase the risk of aspiration and oral residue [14].

Furthermore, PD patients fell in higher impaired trajectories for the variable delayed initiation of the pharyngeal reflex [2]. That finding was not unexpected; similar results have been observed in previous studies using this variable. [2] Various motor disorders of PD have considerable influence on swallowing. For example, disturbed motility in the oral phase of swallowing is characteristic of PD [15, 16]. According to Nilsson et al., the prolongation of the oral-pharyngeal transit time is likely to reflect dysfunction caused by rigidity, bradykinesia (slowness of movement), and hypokinesia [17]. This explanation concurs with the higher impaired trajectories we found for the variable delayed initiation of the pharyngeal reflex in PD. For the other measured FEES variables, PD patients were diversely distributed over the trajectories.

The application of GBTM in an analysis of FEES data is unprecedented, calling for some caution when weighing any new insights it might yield. Under the present circumstances and given the limitations of this study, our conclusions are preliminary and merely indicative. Nonetheless, a few of the interpretations enabled by trajectory clustering are noteworthy, as set forth below.

First, our results lend credence to the hypothesis that to use the *‘one model fits all’* approach, namely to model population trends, leads to an oversimplification of how patients react to the swallow trials. Such techniques may obscure deviations from the norm that are not necessarily linked to known clinical variables. Moreover, analysis of FEES data is often static, focusing on each bolus separately. Note, however, that, if we were to consider each one individually, the detected latent groups would be no longer separable. In our study, trajectories overlapped to a greater or lesser extent during certain parts of the examination. It was only from the developmental perspective that the differences became apparent and here lies the strength of GBTM. The visualization of functional groups may hint at diversity in the nature of change. For instance, for postswallow vallecular pooling, both trajectory nos. 2 and 3 can be considered as lying at an intermediate level of impairment and as being less stable than the other two extremes (upper and bottom trajectories). However, nos. 2 and 3 describe opposite swallowing behaviors. While cluster 2 swallowed better with thin liquids, the transition to thick liquids and a bite-sized cracker was accompanied by a deterioration of the swallowing function. In cluster 3, by contrast, the deglutition of thick liquids and a bite-sized cracker induced substantial improvement in swallowing response of the patients. It is unclear whether these findings reflect different underlying pathophysiological mechanisms affecting the swallowing response (for instance, if trajectory no. 2 would be measuring the effect of fatigue or weakness in the patient or an effect of post radiation xerostomia instead of bolus consistency, contrary to no. 3) [4, 13]. Trajectory no. 2 applied to a larger proportion of HNC patients (post radiation xerostomia). The improvement described by trajectory no. 3 was observed mainly among PD patients and concerned postswallow vallecular pooling with the ingestion of a bite-sized cracker. The DM1 patients fell mainly in the severe impairment trajectory no. 4.

Second, any observed changes in trajectories were most likely induced by the consistency, since points of inflection often coincided with the transitions in the bolus sequence. However, other explanations cannot be disregarded. For instance, the intermediary trajectory no. 2 of postswallow pyriform sinus pooling shows that patients’ swallowing function consistently deteriorated over the swallows, almost independently of the bolus consistencies. Apparently, the subjects fatigued quickly or suffered from severe post radiation xerostomia, leading to a more impaired swallowing function for thick liquid and a bite-sized cracker (mainly HNC patients in trajectory no. 2). Given these results, researchers might do well to adopt a more dynamic approach when analyzing and interpreting the various processes representing patients’ reactive changes to a swallowing challenge. It is only by taking a developmental perspective that patients’ dysfunctional behavior can be better discriminated and the pathophysiology of swallowing impairment properly understood.

The usefulness of GBTM for the analysis of FEES data goes beyond the visualization and characterization of the latent functional groups. Its impact greatly depends on the interpretational value of the trajectories and their theoretical plausibility. As argued here, GBTM provides an insightful depiction of swallowing behaviors. This conclusion needs to be explored further and replicated in larger-scale studies. Clinicians would then have a more detailed evaluation at their disposal, which they could use to support and guide their choices of rehabilitation programs or interventions.

### Limitations of the Study

The present prospective study has some methodological limitations. First, its relatively small sample size posed a major constraint on data analysis with GBTM, as this technique requires larger samples to properly extract latent clusters. Nonetheless, the models converged without difficulty and the indices of model adequacy remained within acceptable bounds. For the analysis of three-point-scale ordinal FEES variables, dichotomization of the measured data may have over-simplified the overall swallow functioning of the patients due to loss of information. Another limitation concerns a well-known misunderstanding linked to GBTM analysis, the *fallacy of reification*. It occurs once latent trajectories are interpreted as real distinct entities. This should be avoided. The intent behind using GBTM was to describe the heterogeneity of FEES outcomes in such a way as to facilitate their clinical interpretation. The identified trajectories are not meant to represent definite classes of swallowing capabilities. The applied FEES protocol in the current study is the standardized protocol we use in daily clinical practice for many years. However, another FEES protocol might produce different results in capturing swallowing trajectories. Finally, a potential drawback of this study is that healthy controls were not included. However, the observed developmental trajectories as described above revealed the presence of clinically relevant subgroups of dysphagic patients in the study population.

## Conclusion

GBTM identified distinct developmental trajectories of FEES responses, capturing the heterogeneity of swallowing function in patients with oropharyngeal dysphagia. These patterns could be linked to the underlying etiology of dysphagia. In clinical practice, such classification of patients into groups may help identify which patients need specific medical attention. GBTM is useful for describing the heterogeneity of dysphagia courses, for identifying groups to determine demographic or biological risk factors, and potentially for informing clinicians about subgroups of dysphagic patients who will need more attention to improve symptom control and quality of life.

## References

[1] Langmore SE, Aviv JE (2001). Endoscopic evaluation and treatment of swallowing disorders.

[2] Baijens LW, Speyer R, Passos VL, Pilz W, van der Kuis J, Haarmans S, Desjardins-Rombouts C (2013). Surface electrical stimulation in dysphagic Parkinson patients: a randomized clinical trial. Laryngoscope.

[3] Pilz W, Baijens LW, Passos VL, Verdonschot R, Wesseling F, Roodenburg N, Faber CG, Kremer B (2014). Swallowing assessment in myotonic dystrophy type 1 using fiberoptic endoscopic evaluation of swallowing (FEES). Neuromuscul Disord.

[4] Nagin DS (2005). Group-based modeling of development.

[5] Nagin DS, Odgers CL (2010). Group-based trajectory modeling in clinical research. Annu Rev Clin Psychol.

[6] Nagin DS, Tremblay RE (2001). Analyzing developmental trajectories of distinct but related behaviors: a group-based method. Psychol Methods.

[7] Shi Q, Mendoza TR, Gunn GB, Wang XS, Rosenthal DI, Cleeland CS (2013). Using group-based trajectory modeling to examine heterogeneity of symptom burden in patients with head and neck cancer undergoing aggressive non-surgical therapy. Qual Life Res.

[8] Pines HA, Gorbach PM, Weiss RE, Shoptaw S, Landovitz RJ, Javanbakht M, Ostrow DG, Stall RD, Plankey M (2014). Sexual risk trajectories among MSM in the United States: implications for pre-exposure prophylaxis delivery. J Acquir Immune Defic Syndr.

[9] Baijens LWJ. Multidisciplinairepolikliniekvoor dysfagie. NederlandsTijdschriftvoor Keel-Neus-Oorheelkunde 18^e^ jaargangnummer 1 januari 2012 p. 5–6.

[10] Folstein MF, Folstein SE, McHugh PR (1975). “Mini-mental state”. A practical method for grading the cognitive state of patients for the clinician. J Psychiatr Res.

[11] Wajsbort J (1997). The, “off-on” phenomenon during treatment of Parkinson’s disease with Levodopa. J Neurol.

[12] Baijens LW, Speyer R, Pilz W, Roodenburg N (2014). FEES protocol derived estimates of sensitivity: aspiration in dysphagic patients. Dysphagia.

[13] Kraaijenga SA, van der Molen L, Jacobi I, Hamming-Vrieze O, Hilgers FJM, van den Brekel MWM (2014). Prospective clinical study on long-term swallowing function and voice quality in advanced head and neck cancer patients treated with concurrent chemoradiotherapy and preventive swallowing exercises. Eur Arch Otorhinolaryngol.

[14] Barbon CE, Steele CM (2014). Efficacy of thickened liquids for eliminating aspiration in head and neck cancer: a systematic review. Otolaryngol Head Neck Surg.

[15] Nagaya M, Teruhiko K, Yamada T, Igata A (1998). Videofluorographic study of swallowing in Parkinson’s disease. Dysphagia.

[16] Bird MR, Woodward MC, Gibson EM, Phyland DJ, Fonda D (1994). Asymptomatic swallowing disorders in elderly patients with Parkinson’s disease: a description of findings on clinical examination and videofluoroscopy in sixteen patients. Age Ageing.

[17] Nilsson H, Ekberg O, Olsson R, Hindfelt B (1996). Quantitative assessment of oral and pharyngeal function in Parkinson’s disease. Dysphagia.

